# An Analysis of Contraceptive Use in Women with a Previous Live Birth in a Single Tertiary Women’s Hospital: A Survey Study

**DOI:** 10.1007/s43032-025-01836-4

**Published:** 2025-02-27

**Authors:** Ye Shen, Hua Fang, Wenmiao Si, Min Zhao, Fangbo Qian, Qi Chen

**Affiliations:** 1 Department of Family Planning, Wuxi Maternal and Child Health Hospital, Wuxi School of Medicine, Jiangnan University, Wuxi, China; 2 Department of Obstetrics, Wuxi Maternal and Child Health Hospital, Wuxi School of Medicine, Jiangnan University, Wuxi, China; 3 Department of Gynaecological Oncology, Wuxi Maternal and Child Health Hospital, Wuxi School of Medicine, Jiangnan University, Wuxi, China; 4 Department of Obstetrics and Gynaecology, Faculty of Medical and Health Sciences, The University of Auckland, Auckland, New Zealand; 5 Department of Obstetrics and Gynaecology, The University of Auckland, Auckland, New Zealand

**Keywords:** Contraceptives, Knowledge of contraception, Survey, Awareness, Education, Promotion

## Abstract

The use of contraceptives to prevent unintended pregnancies is not widely accepted in developing countries due to social and cultural factors, lack of knowledge, and limited access to contraceptive services. This results in a relatively high incidence of repeat abortions in women with live births. The objective of this study is to understand the awareness of contraceptive use in women with live births women with previous live births who currently underwent an abortion (*n* = 272) and women who had a recent live birth (*n* = 323) voluntarily participated in this survey. Among women with previous live births, 22% did not use contraceptives in the past, and 41% well understood the importance of contraceptive use. There were no differences in educational levels, parity, and age at abortion between women who used and did not use contraceptives. However, after the current abortion, a significant increase in women who intend to use contraceptives in the future, from 78 to 93%, was observed. A significant reduction in women who do not intend to use contraceptives from 22 to 7.4% was also observed. 59% of women primarily seek knowledge of contraception from healthcare professionals. Among women with a recent live birth, 92% indicated their awareness of contraceptive use as they do not have a plan for the subsequent pregnancy. More than 91% of women discussed contraceptive use with partners. Our survey highlights the importance of education and promotion of the use of contraceptives in developing countries to prevent unintended pregnancy.

**Supplementary Information:**

The online version contains supplementary material available at 10.1007/s43032-025-01836-4.

## Introduction

Elective surgical abortion is the final option for women who do not want to carry a pregnancy for various reasons, including social and economic factors. Although the immediate potential complications of surgical abortion are relatively few, attention should be paid to the long-term effects on reproductive health. These effects may include secondary infertility, ectopic pregnancy, spontaneous abortion, preterm birth, small for gestational age, and stillbirth in subsequent pregnancy. Multiple induced abortions increase the risk of a subsequent preterm birth, ranging from 25 to 62% [1–3]. Unintended pregnancies could also have negative consequences, such as emotions and economic issues for women and their families. The use of contraceptives not only significantly reduces unintended pregnancies but also provides a fundamental step to prevent potential risks in future pregnancies.

According to the updated World Health Organization (WHO) report, “Family Planning/Contraception Fact Sheet,” published in July 2021, the use of modern contraceptives in women of reproductive age (15 to 49 years) has increased globally [4]. However, there are still gaps in contraceptive use, especially in low-income countries, where many women have limited access to contraceptive services and knowledge or awareness of contraceptive use. The WHO data estimated that 25% of women who want to prevent unintended pregnancy are not using a modern contraceptive method [4]. Furthermore, 17% of women from developing countries who do not want pregnancy are not using any method of contraceptives, which could be the lack of knowledge of modern contraception [5].

The use of contraceptives in women after childbirth to prevent future unintended pregnancies is widely accepted and common in Western countries. However, in some developing countries, there are some barriers to contraceptive use after childbirth. The use of modern contraceptives among Asian women is lower compared to women in Western countries, and the majority of unintended pregnancies among Asian women result from not using any contraceptives [6]. This limited or non-use of contraceptives can be attributed to various social and cultural barriers, lack of knowledge, and restricted access to healthcare services, including accessing contraceptives [7–9]. Studies have identified specific social and cultural barriers among Chinese women, such as fear of side effects, including weight gain, potential infertility, and discomfort, as well as concerns about being perceived as “bad” or promiscuous for using contraceptives [10, 11]. The level of awareness of contraceptive use varies from country to country and among women of different ages in developing countries. The use of contraceptives after childbirth is particularly important regardless of the number of childbirths because short birth intervals can pose reproductive health risks and emotional stress. Contraceptive use also helps women plan their families and achieve their goals after childbirth, such as completing their education or pursuing their career development.

Recent studies from China reported that the incidence of abortion in women with previous live birth(s) was higher than 60%, with 30% of those women having more than one previous abortion [12, 13]. All these data were consistent with the data reported in New Zealand [14]. These findings suggested a lack of promotion of contraceptive use in women after live birth(s). Therefore, we conducted a survey study to understand better the awareness and barriers to the use of contraceptives in women with previous live birth(s) who had a recent abortion, as well as in women who gave birth within six months. Our findings could provide policymakers and health service providers with information on women’s health.

## Methods

This survey study, including the clinical data collection from women who came to the Family Planning Clinic, was approved by the Ethics Committee of Wuxi Maternity and Child Health Hospital (reference number: 2023-06-0511-15). The patient informed consent form for participating in this study was waived by the Ethics Committee of Wuxi Maternity and Child Health Hospital.

### Clinical Information Collection on Women With Surgical Abortion

Using the hospital’s electronic database, we first collected clinical information on women who visited our Family Planning Clinic in Wuxi Maternal and Child Health Hospital, Wuxi School of Medicine, Jiangnan University, Wuxi, China, from January 2022 to December 2022. All women included in this study had undergone a surgical abortion for personal reasons, and those women were from Wuxi urban and Wuxi rural regions. The clinical data collected included their age at surgical abortion, parity, and gravidity.

### Survey Conduction

The questionnaire was conducted from January 2023 to May 2023 in Wuxi Maternal and Child Health Hospital, Wuxi, China. The survey included two cohorts: [1] women with previous birth(s) who visited our Family Planning Clinic and underwent a surgical abortion, and [2] women who had given birth within the last six months during the study period. All women were randomly selected and voluntarily participated in the survey. To address the questions about the awareness of contraceptive use after childbirth or abortions, and to reduce future abortion for unintended pregnancy, and to ensure the participants accurately reflect the cultural norms and feel comfortable and respected through the survey process, a team of epidemiologists and clinicians with expertise in Family Planning and a deep understanding of Chinese cultural contexts designed the questionnaire. There is no individual identification in the questionnaire. The details of the questionnaire are summarized in the Supplementary Tables 1 and 2, which primarily included questions related to contraceptive use, knowledge of contraceptive use, parity, gravidity, and other general information. The use of contraceptives was defined as regularly or continuously using one of the following contraceptives: condoms, intrauterine devices (IUDs), oral pills, or a combination of these contraceptive methods. We acknowledge the potential for response bias, where the participants may not answer truthfully due to several factors presented in this survey study.

**Table 1:** Clinical characteristics of women with previous live birth(s) who visited the Family Planning Clinic with a surgical abortion in the year 2022 (n=4,774)

Age at abortion (years, median/range)	33 (18–44)
Parity (number, %)	
*P* = 1 *P* = 2 *P* ≥ 3	2,885 (60%) 1,739 (36%) 150 (4%)
The number of previous abortions (number, %)	
None Once Twice More than three times	1,373 (28.7%) 1,555 (32.6%) 1,160 (24.5%) 686 (10.8%)

### Statistical Analysis

Data on age at the time of visiting the clinic was expressed as median/range. Other data were expressed as numbers and percentages when appropriate. The statistical difference listed in Table 3 was assessed by the chi-square test using GraphPad Prism (version 9.4). *p* < 0.05 was considered significant. Additionally, Z-values were calculated for the statistical analysis of proportions using OpenEpi (Version 3.01). A Z-values greater than 1.96 was considered statistically significant at a 5% significance level (two-tailed) (Source: https://open.maricopa.edu/psy230mm/chapter/chapter-1-z-test-hypothesis-testing/).

**Table 2:** Clinical characteristics of women with previous live birth(s) who underwent an elective abortion and participated in this survey study (n=272)

Age at abortion (years, median/range)	33 (18–49)
Parity (number, %)	
P = 1 P = 2 P ≥ 3	166 (59.9%) 112 (38.4%) 4 (1.7%)
**The number of previous abortions (number, %)**	
None Once Twice More than three times	67 (25.0%) 83 (30.6%) 63 (22.8%) 59 (21.6%)
**Contraceptive use in the past (number, %)**	
Yes No	212 (77.9%) 60 (22.1%)
**Contraceptive use in the future (number, %)**	
Yes No	252 (92.6%) 20 (7.4%)
**Contraceptive methods (number, %) (in women who used contraceptives, n = 212)**	
Condom Condom and IUD IUD only Oral pill and Condom Oral pills and IUD, and Condom Oral pills and IUD Oral pills only	159 (75%) 5 (2.4%) 10 (4 6%) 21 (10%) 6 (3%) 1 (less than 1%) 10 (4.8%)
**Educational level (number, %)**	
High school Undergraduate Postgraduate	131 (48.2%) 134 (49.3%) 7 (2.5%)
**Knowledge of contraceptive use (number, %)**	
Well understand Partially understand Less understand	112 (41.2%) 148(54.4%) 12 (4.4%)
**Discussion of contraceptive use with partners (number, %)**	
Yes No	266 (98%) 6 (2%)

## Results

We first analyzed the clinical information on women who visited the Family Planning Clinic for surgical abortion from January 2022 to December 2022. There were 6,364 women in our Family Planning Clinic in the year 2022. Of them, 1,590 (25%) women did not have any previous live birth(s). 4,774 (75%) women previously had at least one live birth. Of these women with previous live birth(s), 1,373 (28.7%) women did not previously experience any abortion, 1,555 (32.6%) women previously experienced one abortion, and 1,160 (24.5%) previously experienced two abortions and 686 (10.8%) women previously experienced more than three abortions. The clinical information of women with previous live birth(s) who came to our Family Planning Clinic for abortion is summarized in Table 1.

**Table 3:** The comparison of clinical characteristics between women who underwent abortion with previous live birth(s) who use contraceptives and who do not

	Women who used contraceptives (n=212)	Women who did not use contraceptives (n = 60)	P value
**Age at abortion** (years, median/range)	33 (18–44)	32 (20–43)	0.1427
**Parity (number, %)**			
P = 1 P = 2 P ≥ 3	125 (58.9%) 83 (39.2%) 4 (1.9%)	41 (68.3%) 19 (31.7%) 0 (0%)	0.1668
**The number of previous abortion(s) (number, %)**			
None Once Twice More than three times	51 (24.1%) 62 (29.3%) 52 (24.5%) 47 (22.1%)	16 (25%) 21 (35.4%) 11 (18.8%) 12 (20.8%)	0.8214
**Contraceptive use after**	(total 161 women with		
**the last abortion**	repeat abortions)		
**(number, %)**			
Yes No No answer	127 (78.8%) 16 (10%) 18 (11.2%)	N/A	N/A
**Educational levels (number, %)**			
High school Undergraduate Postgraduate	100 (47.2%) 106 (50.0%) 6 (2.8%)	31 (51.6%) 28 (46.7%) 1 (1.7%)	0.5286
**Contraceptive use in the future (number, %)**			
Yes No	205 (96.7%) 7 (3.3%)	47 (78.3%) 13 (21.7%)	**< 0.001**
**Knowledge of contraceptive use**			
**(number, %)** Well understand Partially understand	87 (41.0%) 117 (55.2%) 8 (3.8%)	24 (40.0%) 31 (51.7%) 5 (8.3%)	0.394
Less understand			
**Source of obtaining the**			
**knowledge on**			
**contraception (multiple**			
**answers) (number, %)**			
Professionals and others	195 (76.4%)	25 (41.6%)	**< 0.001**
**(Professionals only)** Internet and Friends	**147 (75%)** 17 (36.3%)	**14 (56%)** 35 (58.4%)	

To understand better how women with previous live birth(s) are aware of the importance of contraception after giving birth, we conducted a survey of women with previous live birth(s) who visited our Family Planning Clinic for a surgical abortion. In this survey study, 272 women with previous live birth(s) answered our questionnaire (Table 2). A significant number of women (212, 77.9%) regularly used contraceptives even though they failed this time (Z-value = 9.216), and 159 (75%) of these women used condoms as a primary contraceptive method. When combined with oral pills, 180 (85%) women used condoms for protection from unintended pregnancy. We also found a significant number of women (205, 75%) with repeat abortions (Z-value = 8.367).

In this survey, we also found that a significant number of women (252, 92.6%) expressed their intention to or continuously use contraceptives in the future if they do not intend to have a pregnancy after the current abortion (Z-value = 14.07). Compared to the current contraceptive use rate of 77.9%, this represents a significant increase in intended future use (92.6% vs. 77.9%, *p* < 0.001). In this survey, we also found that 52% of women had a bachelor’s degree or above and that most women (95.6%) fully or partially knew contraceptive methods, which was significantly higher (Z-value = 15.04). Interestingly, we also found that a significant number of women (266, 98%) discussed the use of contraceptives with their sex partners (Z-value = 15.76).

We then compared the clinical parameters and other information we collected between women who regularly used contraceptives and those who did not (Table 3). There were no differences in age at current abortion, parity, number of previous abortion(s), and women’s education level between women who regularly used contraceptives and those who did not. There were also no differences in knowledge of contraceptive use and sources of obtaining knowledge of contraceptive use.

Among the 60 women who did not previously use contraceptives, a significant increase in those intending to use contraceptives after the current abortion was observed, with 47 women (78.3%, Z-value = 14.07) expressing this intention. However, this increase was still significantly lower compared to women who regularly use contraceptives (96.7% vs. 78.3%, *p* < 0.001). Our survey found that 75% of women who regularly used contraceptives obtained knowledge of contraceptive use from healthcare professionals such as obstetricians. In contrast, only 14 women (56%) who did not regularly use contraceptives obtained an understanding of contraceptive use from healthcare professionals, which was significantly lower (*p* < 0.001, Table 3).

We also asked women with more than one abortion who regularly used contraceptives whether they used contraceptives after their last abortion (*n* = 212). Among the 161 women with a history of more than one abortion, a significant number of women (127, 78.8%) reported using contraceptives after their last abortion, compared to 16 (10%) who did not (Z-value = 7.329). 18 (11.2%) did not answer this question.

We next surveyed women who had given birth within the last six months (ranging from 4 days to 6 months) to investigate their awareness of contraceptive use if they do not have a plan for a subsequent pregnancy. A total of 323 women completed the questionnaire (Table 4). The median age of participants was 30 years, ranging from 19 to 43 years, and 108 (33.4%) women had a history of previous abortions. Although 23 women (7.2%) do not plan to use contraceptives despite not planning a subsequent pregnancy, a significant number of women (300, 92.8%) believed that contraceptive use was essential if they had no plan for subsequent pregnancy soon (Z-value = 15.41). Furthermore, a significant number of women (303, 94%) reported obtaining knowledge/information about contraceptive use women from healthcare professionals (Z-value = 15.75). The majority of women (80%) had bachelor’s degrees or higher, and 91.3% of women discussed or will discuss contraceptive uses with their sex partners.

**Table 4:** The awareness of contraceptive use in women with a recent live birth (*n* = 323)

Age at delivery (year median/range)	30 (19–43)
**Understanding of contraceptive use after**	
**this birth (number, %)**	
Yes No	307 (95.0%) 16 (5.0%)
**Intend to use contraceptives if no plan for**	
**subsequent pregnancy (number, %)**	
Yes No	300 (92.8%) 23 (7.2%)
**Live births (number, %)**	
P = 1 P = 2 P = 3	226 (70.0%) 87 (27.0%) 10 (3%)
**Numbers of previous abortions**	
**(number, %)**	
None Once More than twice	215 (66.6%) 69 (21.4%) 39 (12.0%)
**Educational level (number, %)**	
High school Undergraduate Postgraduate	65 (20.1%) 227 (70.3%) 31 (8.6%)
**Discussion of contraceptive use with sex**	
**partners (number, %)**	
Yes No	295 (91.3%) 28 (8.7%)

## Discussion

The risk factors associated with repeat or multiple abortions have been clearly defined [15]. To reduce unintended pregnancies, unsafe abortions, transmissions of infection between mothers and newborns, and pregnancy-related risk, the use of contraceptives is extremely important. According to the World Health Organization (WHO) 2021 report, while contraceptive use has increased in women of reproductive age in Western countries, it has not seen significant improvement in developing countries, where up to 25% of unintended pregnancies do not use modern contraceptives in developing countries [4].

Recently, a few Chinese studies reported a higher abortion rate in women with previous live births [12, 13]. Consistent with these studies, our hospital 2022 data also reported that 75% of abortions were performed in women with previous live birth(s). To better understand the awareness of contraceptive use in women with previous live birth(s), we conducted a survey. Our survey found that 75% of women had more than one prior abortion, which was significantly higher than that reported in a 2019 New Zealand analysis (46%) [14] and another study reported in 2006 (up to 50%) [16]. Furthermore, 22% of women in our survey did not use contraceptives in the past. While the exact reasons for not using contraceptives are unknown, studies suggested that lack of knowledge, misperceptions, and exaggerated concerns about the safety of contraceptive methods are significant barriers to contraceptive use in developing countries [17]. Cultural and social norms, as well as the limited availability of contraceptives, also contribute to the low use of contraceptives in developing countries [18–21], including in China [10, 11]. In addition, the awareness of self-protection for unintended pregnancy in Chinese women was not strong [21].

In our survey, we compared the characteristics of women who used contraceptives in the past and who did not. There was no correlation between age at the current abortion, parity, the number of previous abortions, educational levels, and knowledge of contraceptive uses. However, we found that the majority of women (75%) who regularly used contraceptives obtained knowledge of contraception from healthcare professionals. In contrast, only 56% of women who did not regularly use contraceptives obtained knowledge of contraception from healthcare professionals, with the internet or friends being the primary sources of information for these women. It could cause a bias in receiving information/knowledge on contraception or the use of contraceptives.

China is a developing country with some traditional cultures that could result in a lack of knowledge of contraception or barriers to contraception [21]. Although 52% of participants in our current survey had a bachelor’s degree or higher, the other 48% still had lower educational levels, resulting in less than half of participants completely understanding or having knowledge of contraception. As a result, healthcare professionals such as obstetricians or midwives are the most trusted resources for obtaining knowledge of contraception.

Our hospital protocol suggests that our healthcare professionals educate women undergoing elective abortion. We provide a booklet with information on the risk of abortion(s) in future pregnancy and the importance of contraceptive use. The booklet al.so includes the details of contraceptive use. In addition, we also have a contraceptive clinic to provide contraceptive use to women if they need to discuss it. As a result, only 7.4% of women with current abortions decide not to use contraceptives in the future, which is significantly reduced from 22% at the time of the current abortion. This improvement was especially seen in women who did not use contraceptives in the past. 47 (78%) women of 60 women who did not use contraceptives in the past decided to use contraceptives in the future after the current abortion. This suggests that healthcare professional education and promotion of awareness of contraceptive use could significantly benefit preventing unintended pregnancy or repeat abortions. The reasons why those 7.4% of women did not intend to use contraceptives after the current abortion are not entirely clear. However, several factors may contribute to their decision. Fear of potential side effects, such as weight gain and perceived risks of future infertility, may deter some women, regardless of their educational level [10, 11]. In addition, while research has shown that contraceptive pills do not directly cause weight gain [22], temporary weight changes associated with contraceptives may reinforce these concerns. Additionally, limited awareness of contraceptive use is likely a significant factor, particularly among women from rural regions of Wuxi and older age groups. This gap in awareness may also stem from the inadequate emphasis on puberty education, including the prevention of unintended pregnancies, in China several decades ago.

Contraceptives commonly include condoms, oral pills, IUDs, and emergency contraception or combined pills. Our survey found that condoms were the most selected contraceptive method for women who used contraceptives in the past or decided to use them in the future.

In this survey, we also asked women who had a live birth within the last six months about their awareness of contraceptive uses if they do not plan a subsequent pregnancy. Intesteringly, 95% of women understood the importance of contraceptive use after this birth, and 93% of women indicated their intention to use contraceptives if they do not plan for a subsequent pregnancy. From this survey, we also found that the majority of women (more than 97%) discussed the use of contraceptives with their sex partners, suggesting sex partners’ involvement in the use of contraceptives is also essential. This further suggests that educating sex partners could be another key factor for preventing unintended pregnancy or repeat abortions. The identified barriers to contraceptive use suggest that there is a need to improve the use of contraceptives through a community-based and culturally acceptable intervention [23].

Recent studies indicated that women with several disorders may experience reduced sexual libido [24, 25], which can lead to infrequent or no sexual activity. This can influence contraceptive use decisions, as women might perceive contraceptives as unnecessary if they are not sexually active on a regular basis. However, this assumption can potentially lead to unintended pregnancies during sporadic sexual activity. Therefore, it is important to use contraceptives regardless the frequency of sexual activity consistently.

There are some limitations to this study that need to be acknowledged. Firstly, all the data collected in this survey were self-reported, which may have caused response biases. Secondly, this survey was conducted in a relatively wealthy city in China, which may not represent areas with different socio-economic backgrounds. The findings of this study could vary in regions with lower economic development. However, due to the limited puberty education in China, the awareness of contraceptive use to prevent unintended pregnancy may be even lower in less westernized regions in China. Thirdly, some women who reported not using contraceptives in the past may have occasionally used them. Women who reported regularly using contraceptives may also have not occasionally used them.

In conclusion, this survey study demonstrated that 75% of abortions were repeat abortions and that more than 20% of women did not use contraceptives for unintended pregnancies. Clinical characteristics did not differ between women who used contraceptives and those who did not. However, after receiving education and promotion on the importance of contraceptive use during their current abortion, the majority of women indicated their intention to use contraceptives if they did not have a plan for a subsequent pregnancy. Our survey also demonstrated that women with current live birth are aware of the importance of contraceptive use. Healthcare professionals are an essential resource for providing contraceptive education and information to women. To improve the reproductive health of women, including reducing unintended pregnancy and unnecessary abortions, our survey highlights the need for policymakers or healthcare practitioners to promote awareness campaigns about contraceptive use and address concerns regarding the side effects of contraceptives in developing countries.

## Electronic Supplementary Material

Below is the link to the electronic supplementary material.

Supplementary Material 1

## Data Availability

The datasets used and/or analyzed during the current study are available from the corresponding author on reasonable request.
