# Supplementary material for: An Analysis of Contraceptive Use in Women with a Previous Live Birth in a Single Tertiary Women’s Hospital: A Survey Study
Source: Reprod Sci. 2025 Feb 27;32(5):1723–9. doi: 10.1007/s43032-025-01836-4 (PMC12041047; doi:10.1007/s43032-025-01836-4)
Supplement: Supplementary file 1 — Supplementary Material 1 [file 43032_2025_1836_MOESM1_ESM.doc]

**Table 1**

Questionnaire for women who underwent a current abortion

1. Age at current abortion
2. Residence
3. How long have you been in this place?
4. Occupation
5. Educational degree
6. One-Child in the family
7. Gravidity
8. Parity
9. Do you regularly use contraceptives? If no go to question 12.
10. What contraceptives did you use?
11. Which contraceptive methods are most safe?
12. Do you intend to use contraceptives after the current abortion?
13. Which contraceptive methods will you use?
14. Do you understand contraceptive methods?
15. Where do you receive the information or knowledge of contraception?
16. Do you have a plan for having a child?
17. Did you use contraceptives after the last abortion (only for women who had repeat abortions)?
18. Do you understand the importance of contraception after the current abortion?
19. Where did you receive information on contraception after the current abortion?
20. Did you discuss the use of contraceptives with your sex partner?

**Table 2**

Questionnaire for women who had a live birth within the last six months

1. Age at current abortion
2. Residence
3. How long have you been in this place?
4. Occupation
5. Educational degree
6. One-Child in the family
7. Gravidity
8. Parity
9. How old is the baby?
10. Do you regularly use contraceptives?
11. What contraceptives did you use?
12. Which contraceptive methods are most safe?
13. Where do you receive the information or knowledge of contraception?
14. Do you intend to use contraceptives after this live birth, if no plan for the next child?
15. Do you understand the importance of contraception after this live birth?
16. Where did you receive information on contraception after this live birth?
17. Did you discuss the use of contraceptives with your sex partner?
